# Neutral quasispecies evolution and the maximal entropy random walk

**DOI:** 10.1126/sciadv.abb2376

**Published:** 2021-04-14

**Authors:** M. Smerlak

**Affiliations:** Max Planck Institute for Mathematics in the Sciences, Leipzig, Germany. Email: smerlak@mis.mpg.de

## Abstract

Even if they have no impact on phenotype, neutral mutations are not equivalent in the eyes of evolution: A robust neutral variant—one which remains functional after further mutations—is more likely to spread in a large, diverse population than a fragile one. Quasispecies theory shows that the equilibrium frequency of a genotype is proportional to its eigenvector centrality in the neutral network. This paper explores the link between the selection for mutational robustness and the navigability of neutral networks. I show that sequences of neutral mutations follow a “maximal entropy random walk,” a canonical Markov chain on graphs with nonlocal, nondiffusive dynamics. I revisit M. Smith’s word-game model of evolution in this light, finding that the likelihood of certain sequences of substitutions can decrease with the population size. These counterintuitive results underscore the fertility of the interface between evolutionary dynamics, information theory, and physics.

## Teaser

In large, genetically diverse populations (e.g., RNA viruses), neutral evolution is no less directional than adaptive evolution.

## INTRODUCTION

Kimura famously championed the view that a large part of all evolutionary change in genomes confer no selective advantage, i.e., molecular evolution is largely neutral (*1*). Initially based on high observed substitution rates (*2*, *3*), this hypothesis is consistent with the prevalence of extended neutral networks—sets of sequences connected by one-point mutations with equivalent phenotype or function (*4*)—in molecular genotype-to-phenotype maps, e.g., in RNA secondary structure (*5*), protein structure (*6*), or transcriptional regulation networks (*7*). While the exact rate of adaptive versus neutral evolution remains under investigation (*8*), neutralism is now widely understood as a central aspect of evolutionary dynamics (*9*). Besides promoting mutational robustness (*10*–*12*), neutral evolution can enhance evolvability by providing access to novel—and possibly fitter—phenotypes (*13*–*15*). In this way, the variation generated by neutral evolution enables the “arrival of the fittest” (*16*) and can facilitate adaptation in new selective environments (*17*).

Because mutations are random events, it is tempting to picture neutral evolution as a simple random walk (SRW) along neutral networks, and this is how it is usually described, both verbally (*4*) and in quantitative studies (*18*–*21*). The defining property of the SRW is its tendency to explore its environment without any preferred direction, thus approaching an equilibrium distribution that is spread throughout genotype space. This intuition underlies e.g., Gavrilets and Gravner’s approach to speciation in terms of percolating networks in genotype space (*19*, *20*), as well as Crutchfield and van Nimwegen’s proposed link between evolutionary dynamics and equilibrium statistical mechanics (*21*); both rely on the assumption that “after a sufficiently long time, the population is equally likely to be at any of the points of the (giant) component” (*20*). In short, these and other studies equate “neutral” with “equivalent” and “neutral evolution” with “diffusion in genotype space.”

The picture of neutral evolution as an aimless, diffusive exploration of genotype networks is appropriate when population sizes are small and mutations are rare, such that evolution reduces to a sequence of well-separated fixation events (*1*). In the opposite limit where many neutral variants coexist within the population at all times—the “quasispecies” regime of evolution—this picture no longer holds. Instead of spreading uniformly within a genotype network, neutral variants tend to concentrate in highly connected regions within neutral networks, an effect known as the “neutral evolution of mutational robustness” (*10*–*12*). Mutational robustness is normally defined as the fraction of functional one-point mutants of a genotype, but the results in (*10*–*12*) show that neutral quasispecies dynamics maximizes a different property: The most frequent genotypes at mutation-selection equilibrium are the ones with the largest eigenvector centrality in the neutral network. Eigenvector centrality is associated with not only high neutral degrees but also high assortativity (*22*) and cannot be assessed solely from the fitness of one-step mutants of a genotype—it is a nonlocal property, which depends on the structure of the whole neutral network (*23*).

The purpose of this paper is to explore the role of mutational robustness in guiding neutral quasispecies evolution when far from mutation-selection equilibrium. It is well known that clonal interference can lead to the loss of beneficial mutations and limits the speed of adaptation in asexual populations (*24*, *25*). I show here that neutral interference—the competition of mutants with identical phenotype but possibly different robustness, a hallmark of quasispecies dynamics—has a similar, but perhaps more counterintuitive, effect on neutral evolution. Instead of allowing a population to explore its neutral network at a faster rate, increasing the mutation rate and/or the size of the population can shut off its access to noncentral structures within the network; in some cases, the increased pressure for mutational robustness in a larger population can reduce the probability that a particular genotype is sampled during an evolutionary trajectory. As it turns out, this localization effect (*26*), which is conceptually and mathematically similar to the quantum interference responsible for Anderson localization in disordered metals (*27*), can be captured with a different kind of random walk model, the “maximal entropy random walk” (*28*).

## RESULTS

### Quasispecies evolution in a holey fitness landscape

We study the neutral evolution of a population of fixed size *N* using the concept of a holey fitness landscapes (*10*, *11*, *20*). In this approximation, a genotype is either fully functional (Wrightian fitness *w* = 1), or it is wholly unviable (*w* = 0). Picturing genotypes as nodes and mutations as edges, a holey landscape defines a heterogeneous graph *G*, which we assume to be strongly connected for simplicity. We denote *A* the adjacency matrix of this graph, $d(x)=\sum_{y\in G}A_{\mathrm{xy}}$ the neutral degree (number of neutral mutants) of a genotype *x*, and μ the genomic mutation rate.

In Kimura’s classical description of neutral evolution (*1*), a neutral mutant has a probability 1/*N* to fix and replace the wild type through genetic drift. Under this process, the entire population performs a SRW within *G*, whence the concept of populations “diffusing in a neutral network” evoked earlier; because the walk’s jump rate does not depend on *N*, this SRW is also the basis for the molecular clock hypothesis. However, in the regime of evolution where the number of new mutants *M* = μ*N* ≫ 1, as in e.g., RNA viruses (*29*), the selection of mutational robustness becomes more important than genetic drift as a driver of evolution (*10*, *30*–*32*). The dynamics of the distribution of viable genotypes *p*_t_(*x*) is then better described by a replicator-mutator (or quasispecies) equation of the form (*10*)

$$p_{t+1}(x)=\frac{1}{{\left\langle w\right\rangle }_{t}}(\mu \frac{A}{L}+(1-\mu )I)p_{t}(x)$$ (1)

where *L* is the total number of possible mutants for each sequence and the population mean fitness ⟨*w*⟩_t_ = μ⟨*d*⟩_t_/*L* + (1 − μ) ensures that *p*_t_(*x*) is normalized in *G*; see (*11*) for an equivalent continuous-time formulation. From Eq. 1, we see that the equilibrium distribution (or mutation-selection balance) *Q*(*x*) must be the eigenvector of *A* with the largest eigenvalue ρ, which coincides with the equilibrium mean neutral degree ⟨*d*⟩_∞_. Both *Q*(*x*)—also known as the eigenvector centrality of *x* in *G*—and ρ depend on the global topology of *G*, implying that the “neutral evolution of mutational robustness” (*10*) in quasispecies evolution is not merely the maximization of neutrality. What quantity, then, does neutral quasispecies evolution maximize, and what does it imply for evolutionary dynamics before mutation-selection equilibrium is reached?

### Equivalence with the maximal entropy random walk

In (*33*), I outlined an approach to study these questions in a general fitness landscape. The basic idea is that, although Eq. 1 does not by itself describe a Markov process in genotype space [for starters, Eq. 1 is not linear in the density *p*_t_(*x*)], it is in fact closely related to one. To see this, we consider the change of variables *q*_t_(*x*) ∝ *Q*(*x*)*p*_t_(*x*), which satisfies the linear equation (see Materials and Methods)

$$\begin{array}{cc} q_{t+1}(x) & =(Rq_{t})(x)=\sum_{y}R_{\mathrm{xy}}q_{t}(y)\;\text{with}\;R_{\mathrm{xy}}\\ & =\frac{Q(x)(\mu A_{\mathrm{xy}}/L+(1-\mu )\delta_{\mathrm{xy}})Q{\left(y\right)}^{-1}}{\mu \rho /L+(1-\mu )} \end{array}$$ (2)

The matrix *R* in Eq. 2 is a (left) stochastic matrix, and its elements *R_xy_* can therefore be interpreted as Markovian transition probabilities for jumps *y* → *x*. As it turns out, the sequence of transitions generated by *R*—the Markov chain with density *q*_t_(*x*)—is nothing but the maximal entropy random walk (MERW) on *G* (see Material and Methods).

The MERW was introduced in the recent physical literature by Burda *et al.* (*28*) and has found applications in complex networks theory, image analysis, and other fields (*34*–*36*). The MERW is a canonical object on a connected graph *G*—as canonical as the SRW, albeit with very different properties. In a nutshell, while a simple random walker is “blind” (or “drunk”) and therefore chooses the next node to visit uniformly at random among nearest neighbors, a maximal-entropy random walker is “curious”: Her transition probabilities are such that the each new step is asymptotically as unexpected as possible, i.e., the MERW maximizes the late-time entropy rate of the process among all stationary stochastic processes on *G*. The MERW can also be viewed as a SRW on the whole genotype space conditioned on never leaving the neutral network (*37*, *38*) or as random walk on *G* biased by the effective potential *U*(*x*) = − 2 log *Q*(*x*) (*33*) (see Materials and Methods).

Somehow paradoxically, the curious walker following a MERW often has a more difficult time exploring a large irregular network than a blind walker: In the presence of disorder, the equilibrium probability of the maximum-entropy random walker is exponentially suppressed outside a small “localization island” (*28*), see fig. S1. Moreover, as illustrated below, the nonequilibrium behavior of the MERW is strongly directional: Because its dynamics is equivalent to a random walk in the potential *U*(*x*) = − 2 log *Q*(*x*), the MERW walker preferentially follows those trajectories, which increase *Q*(*x*). In the context of neutral evolution, this directional motion implies that regions of genotype space away from its central core are much more difficult to navigate than the standard picture based on diffusion or percolation suggests. For a quasispecies to evolve from *x* to *y* in genotype space, it is not enough that there be a connected path from *x* to *y* within their neutral network *G*—neutral evolution requires more than a percolating neutral network.

### An exactly solvable example: The diving board

**Fig. 1:**
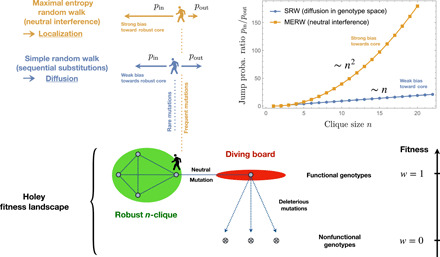
A toy solvable example: The diving board. When mutations are rare and populations are mostly homogeneous, neutral evolution amounts to a SRW along a neutral network of functional genotypes. In larger populations, the interference of multiple neutral mutants leads to the selection for mutational robustness, which can be described as a MERW on that network. The qualitative difference between the SRW and the MERW is illustrated here by a simple network configuration, where an *n*-clique is connected to a degree-one node or diving board. In that case, the SRW on the edge of the clique will jump back into it with probability *p*_in_ ∼ *np*_out_, while the MERW will jump back with the much higher probability *p*_in_ ∼ *n*^2^*p*_out_. Similar effects can induce the localization of populations within neutral networks, invalidating commonly analogies between neutral evolution and diffusion (or percolation) in sequence space.

Basic intuition for the difference between the SRW and the MERW can be gained through an elementary example (Fig. 1). Consider a fully connected graph with *n* nodes (an *n*-clique) with one extra node attached (a “diving board”). (This configuration is not biological, because Hamming graphs do not contain these cliques. The point is merely to offer an analytically solvable example.). We wish to compare the probability that a walker on the edge of the clique jumps back into it (denoted *p*_in_) with the probability that it steps onto the diving board (denoted *p*_out_). Because there are *n* − 1 edges going in and 1 edge going out, for the SRW, these probabilities are simply $p_{\text{in}}^{\text{SRW}}=(n-1)/n$ and $p_{\text{out}}^{\text{SRW}}=1/n$; hence, $p_{\text{in}}^{\text{SRW}}/p_{\text{out}}^{\text{SRW}}=n-1$.

To evaluate the same ratio for the MERW, we must compute the dominant eigenvector *Q* of the adjacency matrix *A* for the complete graph over *n* nodes {1, ⋯, *n*} with one extra node, labeled 0, attached to vertex 1. By symmetry, this eigenvector *Q* = (*q*_0_, *q*_1_, ⋯*q_n_*) can be chosen such that *q*_2_ = ⋯ = *q_n_* = 1. Writing *AQ* = ρ*Q* then gives a system of two quadratic equations for (*q*_0_, *q*_1_), from which we then obtain $p_{\text{in}}^{\text{MERW}}/p_{\text{in}}^{\text{MERW}}=(n-1)/q_{0}$. Because *q*_0_ ∼ 1/*n* when *n* ≫ 1, this gives $p_{\text{in}}^{\text{MERW}}/p_{\text{out}}^{\text{MERW}}∼n^{2}$ as plotted in Fig. 1. Thus, in this configuration, the MERW walker is much more strongly attracted by a large clique than the SRW walker. Because the MERW is directly related to neutral quasispecies evolution, we see that the topology of neutral networks affects not only the mutation-selection equilibrium but also the kinds of evolutionary trajectories, which are likely to be realized.

The behavior observed in the diving board example reflects the information-theoretic optimality of the MERW: Because there are many more ways into the clique than out of it, the walker generates more information (more “surprise”) by selecting an inward path than by following the unidirectional ridge. The transition probabilities in Eq. 2 and defining the MERW are precisely those that maximize the entropy rate along paths of length *T* → ∞ (*28*, *39*). In that sense, we can say that quasispecies evolution is directed toward those regions of genotype space where neutral mutation will be generated with the maximal possible information rate.

### Revisiting Maynard Smith’s four-letter model

**Fig. 2:**
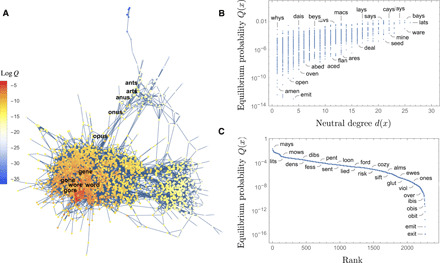
Localization in Maynard Smith’s holey landscape of meaningful four-letter words. (**A**) The giant component with vertices colored by their logarithmic probability at mutation-selection balance, with the neutrals paths σ and σ′ highlighted in boldface characters. While σ is deep in the core and easily evolvable, σ′ belongs to a narrow ridge, which can hardly be traversed. (**B**) The evolutionary stability of four-letter words, measured by their probability at mutation-selection equilibrium, is correlated with their neutral degree (mutational robustness) but cannot be reliably inferred from it. (**C**) The rank plot of *Q*(*x*) shows the approximately exponential decay of the equilibrium density away from the core typical of localization phenomena.

To illustrate the implications of this dynamical property, I reconsidered Maynard Smith’s famous toy model of protein evolution (*4*). In the set of all possible four-letter words, Maynard Smith used meaning as a binary measure of fitness: All meaningful words are functional, and all meaningless ones are nonfunctional. He then gave the sequence of one-point mutations σ = (WORD, WORE, GORE, GONE, GENE) as an example of a neutral path, arguing that, unless a large section of genotype space can be traversed through these paths, molecular evolution is impossible. (Maynard Smith’s general argument includes both neutral and adaptive paths; his word-game example and this paper focus on the former.) It is easy to come up with other examples of neutral paths; in the following, we will focus on σ ′ = (OPUS, ONUS, ANUS, ANTS, ARTS), which lies on a ridge within the neutral network of meaningful words (Fig. 2A).

Using the Wolfram dataset of English “KnownWords,” we find that of 2405 meaningful four-letter words, 2268 belong to the giant component *G*, including both paths σ and σ′. However, due to the irregular structure of *G* with three communities separated by narrow ridges (Fig. 2A), most of these words have negligible equilibrium probability: A core of just 420 (respectively 1064) words concentrates 90% (respectively 99%) of the total probability (Fig. 2C and fig. S2). In particular, if all the words in Maynard Smith’s path σ (except GENE) belong to the 99% core, none of the words in σ′ do. Note that the words with the largest equilibrium probability are poorly predicted from their neutral degree (Fig. 2B): While SAYS and SEED both have relatively high neutrality (*d* = 21), the former is 10,000 times more frequent than the latter. This highlights that the “neutral evolution of mutational robustness” is not simply the evolutionary advantage of robust genotypes—it is a selection principle, which singles out, on a logarithmic scale, a subset of robust (e.g., BAYS) and nonrobust (e.g., WHYS) genotypes that are globally well connected in *G*. These patterns generalize to words of different lengths (Table 1 and fig. S4) and conform to a recent observation of Altenberg (*23*).

**Table 1:** Holey landscapes of meaningful English words of different lengths (see fig. S4).

**Word length**	**Number of meaningful** **words**	**Giant component size |*G*|**	**Giant component effective** **size |*G*|_eff_**	**Size ratios |*G*|_eff_/|*G*|**
3	621	603	265.4	0.44
4	2403	2268	346.6	0.15
5	4753	3598	588.9	0.16
6	7763	3353	64.9	0.02
7	10926	2081	42.67	0.02

**Fig. 3:**
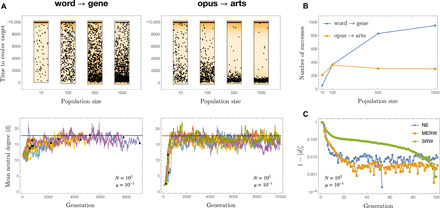
Localization in the neutral network of short RNA secondary structure. (**A**) The evolution of ARTS from OPUS (left column) is a very different challenge than that of gene from WORD (right column), as is seen by comparing the time to evolve the target word at varying *N* (top row, with a cutoff after 10^4^ generations) and the shape of ⟨*d*⟩_t_ trajectories at *N* = 10^3^ (bottom row, with the black horizontal line representing the equilibrium value ρ and black dots the endpoint of successful trajectories). While the likelihood of success of WORD → GENE increases with the population size, it does not for OPUS → ARTS. If a population does not succeed to evolve ARTS in the first few generations of the run before it can get trapped in the core, then it never will. (**B**) Instead of growing linearly with *N*, the likelihood to evolve a genotype from another through neutral evolution can depend nonmonotonically on the population size. Here, there were 10^3^ attempts with μ = 0.1, and a population with 100 individuals was more likely to evolve OPUS → ARTS at least once than one with 1000 individuals. (**C**) The convergence to equilibrium, here measured by the rescaled mean degree ⟨*d*⟩_t_′, is much faster in a neutrally evolving population (NE) than in diffusing one (SRW) of the same size; it is however consistent with the fast mixing property of the MERW (*40*).

In multiple runs of a simple evolutionary simulation with a 10% mutation probability per genome per generation, population size *N* = 10^3^, and time horizon of 10^4^ generations, GENE almost always evolved from WORD (through Maynard Smith’s or, more often, some other path), but—because of the fragility of its intermediate forms and its noncentral location in the network—ARTS only rarely evolved from OPUS (Fig. 3A and fig. S3). When it did evolve, ARTS arose in the first few generations of the run before the population got permanently trapped in the core. Smaller (but not too small) populations had a higher probability to evolve ARTS at least once within the prescribed time horizon (Fig. 3B). This effect is due to the coupling between individuals through negative selective pressures and would be incomprehensible if we viewed neutral evolution as diffusion (in which case the success rate would grow linearly with *N*). Last, we observe that neutral evolution converges faster to the equilibrium distribution *Q*(*x*) than the SRW (Fig. 3C), consistently with the fast mixing property of the MERW (*40*); in Fig. 3C, this convergence is represented in terms of the rescaled mean degree ⟨*d*⟩′_t_ = (⟨*d*⟩_t_ − min_s_⟨*d*⟩_s_)/(max_s_⟨*d*⟩_s_ − min_s_⟨*d*⟩_s_).

### A small RNA secondary structure

**Fig. 4:**
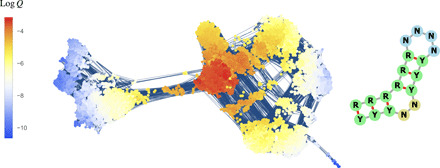
Localization in the neutral network of short RNA secondary structure. The neutral network of the RNA secondary structure “((((((….)))..)))” with only purine (R)-pyrimidine (Y) base pairs (*10*), as predicted by the RNA Vienna algorithm v2.4.8. Its giant component consists of multiple communities separated by narrow ridges, which induce the localization of the mutation-selection equilibrium *Q* and limits nonequilibrium dynamics accordingly. For this reason, the notion that diffusion enables the search of vast areas in genotype space (*18*) must be understood as holding within communities but not necessarily between them.

As a second illustrative example, I reconsidered the neutral network of RNA secondary structures studied in (*10*, *41*). The secondary structure of an RNA molecule is the pattern of pairings between complementary bases along its sequence and can be represented with brackets (paired bases) and dots (unpaired bases). Here, we consider sequences of length 𝓁 = 18 with minimum-free–energy secondary structure **((((((….)))..)))** and only purine-pyrimidine base pairs. With the RNA Vienna folding algorithm v2.4.8 (*42*), the giant component has size ∣*G* ∣ = 17,557, but its mutation-selection equilibrium distribution *Q*(*x*) varies over seven orders of magnitude between core and periphery due to narrow ridges within *G* (Fig. 4); these ridges are also seen in the experimental assay of a small protein neutral network (*43*) and may be a generic feature of biological genotype-to-phenotype mappings (*44*). Thus, in molecular evolution and in Maynard Smith’s toy model, neutral evolution may not efficiently sample neutral networks. Statements to the effect that “diffusion enables the search of vast areas in genotype space” (*18*) must therefore be qualified accordingly. For a related discussion in the weak-mutation regime (*M* ≪ 1), see (*41*).

### Robustness and evolvability

The association of neutral evolution with the MERW rather than the SRW has notable implications for the classical issues of robustness and evolvability (*45*). In the literature, the potential of (e.g., RNA) sequences to generate new phenotypes—their evolvability—has been related to the (linear or logarithmic) size ∣*G*∣ of their neutral network (*46*), also known as their versatility. The rationale for this hypothesis is that a large neutral network potentially communicates with many other neutral networks (other phenotypes) through “portal” sequences interfacing between them. However, just like the randomness of an information source should be quantified by its entropy and not merely its alphabet size (*39*), the reach of neutral evolution should be quantified by its ability to generate new sequences, not by the number of all possible sequences. Information theory provides the correct language for this: In the *M* ≫ 1 regime, the versatility of a phenotype should be measured by the entropy $H(Q)=-\sum_{x\in G}Q(x)\text{log}Q(x)$ of its mutation-selection equilibrium or equivalently by the effective size ∣*G*∣_eff_ = 2^*H*(*Q*)^ of the giant component. In both cases considered above, this quantity is much smaller than the naive value ∣*G*∣: With four-letter words, we have ∣*G*∣_eff_ = 346.6 ≪ 2268, and for the RNA secondary structure, ∣*G*∣_eff_ = 3971.8 ≪ 17,557. This compression affects the evolvability of the structure: While there are 357 distinct structures in the one-point neighborhood of the giant component *G*, only 198 of them can be accessed by mutations of sequences in the core where 90% of the equilibrium probability lies.

## DISCUSSION

I have described the localization of populations within neutral networks induced by the competition for mutational robustness among neutral mutants. This “neutral interference” is interference in the two senses of the word: In the biological sense, because it involves the competition of clonal subpopulations, an effect usually referred to as clonal interference; and in the physical sense, because localization is an interference phenomenon normally encountered in (classical or quantum) wave mechanics. The link between these two seemingly different processes—the evolution of molecular populations and the destructive interference of waves—is provided by the MERW, a canonical Markov chain whose transition probabilities depend on the global structure of the underlying graph. For a more extensive discussion of these analogies, I refer the reader to (*33*, *47*).

These observations reveal sharper constraints on the navigability of neutral networks than previously appreciated: Not only does evolution favor high mutational robustness and low genetic loads, but it also positively refuses to explore structures that do not point to the high-centrality core of the neutral network. This effect manifests itself in the surprising nonmonotonic dependence of discovery rates of certain target genotypes on population size and implies that hopes to infer the complete structure of neutral networks from measurements of transient population dynamics (*10*) are unfounded. In this way, we can only hope to infer the structure of the robust cores of neutral networks. In large molecular or viral populations, neutral evolution is no less directional than adaptive evolution.

## MATERIALS AND METHODS

### Robustness and evolvability

In (*33*), I showed that continuous-time replicator-mutator (or quasispecies) equations can be understood in terms of a derived Markov process, for which the logarithm of the selection-mutation equilibrium plays the role of an effective potential. With discrete generations, this scheme can be reformulated as follows: Given a discrete space *X*, consider a sequence of probability distributions *p_t_* : *X* → ℝ evolving under the dynamics

$$p_{t+1}(x)=\frac{\left(Bp_{t})(x\right)}{\sum_{y}(Bp_{t})(y)}$$ (3)

with *B* any irreducible nonnegative matrix. (Here, I identify a function on *X* with the vector of its values.) In replicator-mutator systems, we have *B* = *MW*, with *W* as a diagonal matrix of Wrightian fitnesses and *M* as a stochastic matrix of mutation probabilities. The process defined by Eq. 3 is not a Markov process due to the global interactions introduced by the normalization factor; in particular, Eq. 3 is not readily interpretable in terms of “evolutionary trajectories.” This can be remedied by means of the change of variable *q*_t_(*x*) ∝ *S*(*x*)*p*_t_(*x*), where *S* is the (left) eigenvector of *B* with largest eigenvalue; by the Perron-Frobenius theorem, this vector is positive and its eigenvalue is the spectral radius σ of *S*. Via this transformation, which only depends on *t* through a global constant, we obtain a Markovian representation of the original dynamical problem with master equation

$$q_{t+1}(x)=\tilde{B}q_{t}(x)\;\text{with}\;{\tilde{B}}_{\mathrm{xy}}=\frac{S(x)B_{\mathrm{xy}}S{\left(y\right)}^{-1}}{\sigma }$$ (4)

For this derived process, the function *U* = − 2 log *S* plays the role of a potential; its analysis reveals the metastable states and preferred trajectories of the original (nonlinear) process (*33*, *47*). The equilibrium distribution for Eq. 4 is *q*_∞_(*x*) ∝ *S*^2^(*x*), and the original distribution can be reconstructed from *q*_t_(*x*) with *p*_t_(*x*) ∝ *S*(*x*)^−1^*q*_t_(*x*). When *B* happens to be the adjacency matrix *A* of a connected graph *G*, as in neutral evolution, the process Eq. 4 coincides with the MERW on *G*.

### The maximal entropy random walk

The SRW on a connected graph *G* with adjacency matrix *A* and degree matrix *D* = *diag*(*d*(*x*))_x ∈ G_ is the Markov chain with transition matrix

$$R_{\mathrm{xy}}=\text{Prob}(x|y)=\frac{A_{\mathrm{xy}}}{d(y)}$$

By contrast, the MERW was defined in (*28*) as the Markov chain with transition matrix

$$\text{Prob}(x|y)=\frac{Q(x)A_{\mathrm{xy}}Q{\left(y\right)}^{-1}}{\rho }$$

where *Q* is the dominant eigenvector of *A* with eigenvalue ρ (equal to the spectral radius of *A*). This Markov chain maximizes the entropy rate among ergodic stationary processes on *G* by assigning equal probability to all paths of fixed length connecting two given nodes. Rather paradoxically, this property leads to localization when the graph *G* is irregular, as illustrated in fig. S1.

## SUPPLEMENTARY MATERIALS

Supplementary material for this article is available at http://advances.sciencemag.org/cgi/content/full/7/16/eabb2376/DC1

## Appendix

View/request a protocol for this paper from *Bio-protocol*.
